# To be a trained and supported volunteer in palliative care – a phenomenological study

**DOI:** 10.1186/s12904-017-0193-0

**Published:** 2017-03-14

**Authors:** Ulrika Söderhamn, Sylvi Flateland, Marthe Fensli, Ragnhild Skaar

**Affiliations:** Centre for Care Research, Southern Norway, Faculty of Health and Sport Sciences, Campus Grimstad, University of Agder, Post Box 422, 4604, Kristiansand, Norway

**Keywords:** Benefits, Challenges, Clarified role, Community health care services, Followed-up, Meaningful experience, Phenomenology, Presence in volunteering, Qualitative research, Seriously ill

## Abstract

**Background:**

It has been found that including volunteers in palliative care is a positive contribution to seriously ill patients. It is, however, recommended that the volunteers are trained and supported. The aim of this study was to describe a group of trained and supported volunteers’ lived experiences as volunteers in palliative care within the community health care services.

**Methods:**

This study adopted a descriptive phenomenological approach featuring individual interviews with nine volunteers. The interviews were analysed using the descriptive phenomenological research method according to Giorgi.

**Results:**

Being a volunteer in palliative care was both a positive and meaningful experience. It was a privilege being able to help those in need, which yielded positive returns. As a volunteer, it was important to be present for the ill persons and to follow them in their various physical and psychical states, which also implied that the volunteer had to face and deal with challenging situations. However, volunteers stated it was crucial to possess knowledge and life experience, as well as a clarified role, and they stressed the importance of being followed up by a mentor.

**Conclusions:**

The findings showed that trained and supported volunteers among seriously ill or dying people within the realm of community health care services play an independent and important role in the palliative care team. A coordinator in palliative care is especially suitable for training and supporting the volunteers.

## Background

The inclusion of volunteers in palliative care was found to be a positive contribution to seriously ill patients [1] and their families by improving their well-being. It has even been reported that palliative patients who received visits by volunteers have longer survival periods than those who did not receive these visits [2]. However, it is recommended that the volunteers be trained and supported in their volunteer activities, which is also consistent with volunteers’ wishes [3]. In Norway, the government has stated that the best care is provided through close cooperation between the public health care services, the patients and their families, and the volunteer care providers [4]. Since it can be challenging to be a volunteer in palliative care, the Norwegian government also states that volunteers should perform customized tasks and be monitored through training and guidance [5]. This study will focus on trained and supported volunteers in Norway, as well as on their experiences of volunteering among seriously ill or dying people in palliative care in the community health care services.

The role of the volunteer in palliative care is different when compared with the role of paid staff members. Volunteers are described as complementary [1], since they can provide practical assistance [6]. When a volunteer undertakes a task that is not being performed by anyone else on the team caring for the patient, the role is described as independent. The volunteer role is also referred to in terms of surrogacy, as the volunteer can even replace a family member in some instances [1]. An important volunteer task in palliative care is to provide emotional [1, 2] and social support [1, 2, 7], as well as companionship [2, 6], which is connected to the idea that the volunteer can become a friend to the patient [2]. However, the tasks that a volunteer performs can vary, especially since certain tasks often depend on what is being requested by the patient and his/her family members [1]; in this way, the volunteer plays a unique role that intermediates between the patient, the family, and staff members [2].

Many volunteers in palliative care have a desire to become volunteers because they have had experiences with serious illness and death in their families. Some individuals choose to become volunteers because they wish to help and care for suffering patients [7] while making a difference for them [6]. Those who are volunteers in palliative care have been found to possess such personal characteristics as agreeableness, extraversion, being empathetic, and being emotionally robust [3]. Their volunteering provided them with benefits such as personal gain [3, 8], growth [6], satisfaction [9], and an understanding of what is important in life [6].

Being a volunteer in palliative care is known to include a lot of stress. Examples of reported stress factors have been a lack of support, poor communication, and dealing with death. Volunteers have also experienced their role as ambiguous, particularly due to the lack of clarity related to their tasks. However, they have also experienced themselves as flexible because they have to perform tasks that no one else has the time to do [3]. Their relationship with staff members is known to differ, and can range between feeling as though they are part of the staff’s team to being undervalued [10] and controlled [1]. Likewise, encounters with a patient’s family can be experienced as challenging [11].

Those volunteers who report feeling satisfied with their volunteering activities tend to be those who were provided with adequate training and support. This feeling propels them to continue their voluntary work. A volunteer coordinator thus holds an important role in training and supporting the volunteers [10]. It is of crucial importance that the volunteers want to continue to service their community, particularly since there is supposed to be a future shortage of volunteers [4]. However, in Scandinavia, there is a paucity of studies on volunteers’ experiences while volunteering in palliative care, and there is also a shortage of studies conducted among volunteers who are trained and supported by a palliative care coordinator.

## Methods

### Aim

The aim of this study was to describe a group of trained and supported volunteers’ lived experiences as volunteers in palliative care within the community health care services. This study seeks to answer the following research question: What are volunteers’ experiences of “being a trained and supported volunteer in palliative care” among seriously ill or dying people?

### Design

This study adopted a descriptive phenomenological approach using individual interviews conducted with volunteers in palliative care.

In phenomenology, one has to be present to observe how a phenomenon is experienced by the consciousness of the experiencer, and that individual must describe it as precisely as possible, i.e., neither adding nor subtracting anything from what is experienced. Thus, phenomenology can be described as the study of a phenomenon’s structure and variations of this structure, i.e., how the phenomenon is perceived and how it appears in various people’s consciousness. However, in order to be able to describe the narrated lived experiences as they are presented, one has to bracket his or her own knowledge about the phenomenon [12].

### Study group and setting

Volunteers working among seriously ill or dying persons in the health care services within a municipality in the southern part of Norway were requested to participate in the study. Ten individuals met the inclusion criteria: 1) individuals who were currently serving as volunteers and who were required to interact with people with a serious illness in the palliative phase (who were either living in their own homes or at a nursing home), and/or 2) individuals who were serving as volunteers and who were responsible for holding a 2-h group meeting every second week for seriously ill individuals with cancer, and 3) volunteers that were not connected to any association, such as the Red Cross.

The palliative care coordinator approached the ten eligible volunteers about their participation in the study, and they were provided with verbal and written information about the study. All of the volunteers showed interest in participating in the study, and they provided their permission to receive a telephone call from the first author to obtain more information about the study; thereafter, all of the participants agreed to participate in the study, and appointments for interviews were made. However, one chose to withdraw before the interview was conducted. Thus, nine volunteers (six women and three men) were included in the study.

In terms of age, the volunteers were in their mid-twenties to mid-seventies, and six volunteers were retired. Six of the volunteers had prior work experience in the social or health care sectors, while two had experience within the school sector and one had another work experience. Some volunteers also had the experience of living with cancer, or they had family members who had been seriously ill and passed away.

Their total experience as volunteers in palliative care varied between 6 months and 7 years. Some of these individuals were handpicked to become volunteers, while some had expressed their interest to become volunteers in palliative care. They worked as volunteers from 3 to 4 h per week to 2 h every second or third week, and they had been volunteering among seriously ill or dying persons in their home (four volunteers) or at a nursing home (three volunteers), or they had led group meetings for serious ill people with cancer (five volunteers).

Before volunteering, the volunteers were provided with training, which included attending a course that was adapted to their competence and experience, to prepare them for voluntary work among seriously ill or dying persons. The palliative care coordinator led the course. The following themes were emphasized: to be a volunteer, to be seriously ill, people in crisis, to go into someone else’s home, to face death and sorrow, communication, and confidentiality. In addition, twice a year, the coordinator invited the volunteers to a meeting that featured a lesson on a current theme, and where everyone could discuss and reflect on their experiences. The coordinator served as a mentor for all of the volunteers, and they were regularly followed-up with and debriefed after their volunteer sessions. The volunteers could send an SMS after each volunteer session, and they were able to write how their volunteering experiences went, and they could indicate if they wished to talk about their experiences or not. The mentor was available by telephone each day and evening. All of the volunteers had signed an agreement regarding their volunteer activities (which was renewed every year), and they signed a confidentiality agreement.

The palliative care coordinator was responsible for choosing the right volunteer for each seriously ill person in need of help, and the coordinator was also in charge of selecting volunteers to lead group meetings for individuals with cancer. Those informants who volunteered with seriously ill persons in their home provided social support, practical help, and activities such as walking tours, all of which depended on the ill person’s status. In this way, the volunteers also provided relief and respite for the ill person’s relatives. In nursing home settings, the volunteers provided social support. In the group meetings, two volunteers worked together and they chose and organized a program for the meetings; they also chose a theme for the day to discuss with the ill individuals. Moreover, they read poems or other texts that addressed the theme, they ate together and, when needed, they invited health care professionals to inform group participants about actual disease-related themes. The palliative care coordinator was responsible for making practical arrangements regarding the group meetings, and assisted the volunteers when needed.

### Data collection

All interviews were conducted between February and March 2015. Eight of the interviews took place at the local university and one interview was held at an informant’s workplace. The informants were asked to talk freely about their experiences as a volunteer among seriously ill persons. When doing so, they were encouraged to talk about a positive experience and a challenging experience as a volunteer. Follow-up questions such as “Can you tell me more about that?”, “Can you explain it further?”, and “What did it mean for you?” were asked to obtain richer descriptions. The interviews lasted up to 50 min and they were recorded and transcribed verbatim.

### Analysis

The analysis of the interviews took place within the perspective of phenomenological scientific reduction, i.e., the researcher’s knowledge about the phenomenon was bracketed. Georgi’s [12] descriptive phenomenological research method was used, and the analysis adhered to the following steps: 1) *Read the whole interview text to obtain a general sense of what the text is about*. 2) *Determine meaning units*. After re-reading the interview text, the description was broken down into parts where there was a shift in meaning in the text. This was done to make descriptions more manageable. 3) *Transform the informants’ natural attitude expressions into health science language*. By going back to the beginning of the interview text, each meaning unit that was originally expressed in the informant’s own words was transformed using a phenomenological procedure of free imaginative variation. This involves imaginatively varying the data in the meaning units until one finds the most suitable expression without losing the informant’s expressed meaning. The transformed meaning units were then synthesized into a situated structure (a consistent statement for each informant) expressing the phenomenon of volunteering in palliative care. 4) *Write a general structure*. A general structure pertaining to the phenomenon of “being a trained and supported volunteer in palliative care” was written. Transformed meaning units and common features in the situated structures formed the basis of writing this general experience structure. The general structure is the identification of the constituents that are essential for the phenomenon to manifest itself, and it illustrates how these constituents relate to each other.

The analysis revealed a general structure with six invariant features, which is presented in the Findings section.

## Results

### The general structure

Being a volunteer among seriously ill persons was both a positive and meaningful experience. It was a privilege being able to help those in need, which yielded positive returns. As a volunteer, it was important to be present for the ill persons and to follow them in their various physical and psychical states, which also implied that the volunteer had to face and deal with challenging situations. However, volunteers stated it was crucial to possess knowledge and life experience, as well as a clarified role, and they stressed the importance of being followed up by a mentor.

### A privilege being a volunteer

All of the informants found that being a volunteer was a positive and meaningful experience that provided a sense of happiness and peace, and a feeling of being useful. Thus, it was described as a privilege to be a volunteer, i.e., a fellow man, and being able to help the ill person. It was important and satisfying to mean something to someone who was struggling, and to be able to make a difference for this person. Moreover, it was enriching to obtain insight into the lives of the ill individuals and to understand the different ways in which these individuals handled their diseases and their life situations. This led to the idea that the volunteers learned a lot and were grateful for their own lives. They regarded their volunteer experience as satisfying, and they wished that more people would want to become volunteers in palliative care. However, it was emphasized that in order to engage as a volunteer, one must be interested in volunteering and they must also have the desire and energy to help others. Some also expressed that as a volunteer, one cannot have mental problems.

Those volunteers who visited the ill persons in their homes found it surprising that the volunteers were well received from the beginning; moreover, the volunteers were shown trust despite the fact that the volunteers were unknown to the ill individuals. The volunteers responsible for the group meetings experienced that the group participants shared their experiences in an open and honest way. It was encouraging for the volunteers to receive feedback from the participants, in that the meetings were perceived as useful and meaningful.

All of the volunteers experienced that their volunteering provided many positive gains; for example, they were appreciated and received positive feedback and confirmation about their volunteering from the ill persons and/or from the ill persons’ families. The volunteers found that they received more than they gave in their volunteering initiatives. Another gain was found insofar as the volunteers provided relief and respite for the relatives; moreover, they were also able to help family members cope with a loved one’s illness and death. These gains were motivating factors that encouraged them to continue their volunteering. Some of the volunteers expressed that they felt like egoists because they continued to volunteer due to the positive feedback they received.

### To be present

Throughout their volunteer experiences, all of the volunteers indicated that they prioritized being present for the ill person. They had the time and opportunity to direct their full attention towards the person, which is in contrast with health care professionals who must take care of many patients simultaneously. It was considered important to see and treat the ill persons with respect and to take them seriously. To see the person behind the illness, to be able to detect the ill person’s needs, and to identify what the volunteers themselves could do provided a feeling of closeness and connection between the ill person and the volunteer. Thus, the volunteers succeeded at being present for the other. To achieve this aim, one of the volunteers stated how it helped to look at pictures or photo albums, or to ask the relatives about the ill person’s various interests.

All of the volunteers indicated that their presence should be compassionate, humble, patient, and affirmative, while listening and being open to the ill person’s needs. It was also described that the volunteers had to be confident, where they could hold eye contact and be a good listener. They should not talk too much, but rather actively listen and ask questions by grasping what the ill person said. Sometimes words were not necessary. To be receptive to what the ill persons wanted to talk about was a way to follow them in their physical and psychical states. As a volunteer, they had to let the ill persons talk about their own frustrations, but the volunteers also had to act as supervisors who provided information and advice. Apropos the supervisor function, one volunteer mentioned that the ill persons and their relatives often ask questions that, unbeknownst to them, they already know the answers to. The volunteer stated how she/he has to help these ill persons or their relatives come to realize that they do, in fact, possess the answers.

The volunteers who visited the ill persons in their homes or in the nursing home found it important to become close with these individuals; this could be achieved by holding the ill person’s hand, giving that person a hug, holding an arm around the person’s shoulders, or crying together. They got to know the ill persons very well, and the volunteers could see whether an individual had something to say, so the volunteer could help that individual formulate a difficult issue. Sometimes the volunteer had to ask difficult questions, especially regarding death, and to give honest answers. The volunteer could be the one person who the ill persons wanted to talk to about difficult issues; the ill individuals felt they could not talk to others. As volunteers, they also considered it important to be conscious of their own faith and to show respect for individuals of another faith when talking about death and life after death. Some of the volunteers had the opportunity to follow the ill person to the end, and they were also able to attend the funeral. If it was not possible to attend the funeral, the volunteers could end their service by visiting the relatives afterwards.

Those volunteers who were responsible for holding the group meeting program deemed it important to meet the group’s wishes and to be present and become close with the participants. The theme for the meeting could, for example, be about how to handle bad days or how to meet each other during a life crisis. To achieve balance in the program, the volunteers also wanted to focus on quality of life, the joy of life, and social activities. They attempted to find good stories that they could read, stories that connected with the theme at the meeting. In subsequent talks with the group participants, the volunteers needed to have the courage to be honest and giving of themselves; however, it was emphasized that the group meeting should be a resting place and a breathing space for the participants. It was considered important to see each of them and to give those who wanted to talk the confidence to talk, as well as to help them articulate their feelings; it was also important to clarify that there was no requirement to talk. The volunteers had to be aware not to stigmatize those living with a disease. Instead, they had to be present, while supporting and strengthening them. This could mean that they sometimes all laughed and cried together. As one of the volunteers said, to be a volunteer in this group, you have to dare to cry.

### To face and deal with challenges

All of the volunteers had experienced challenges when volunteering. Their voluntary work could be difficult at times, as they faced several tough situations, such seeing a person suffer or watching a young person die. It was painful to experience the reactions that the terminally ill persons and/or their relatives could exhibit at the end of life. However, none of the volunteers characterized such experiences as negative; instead, they found these experiences to be natural, and they wanted to be present and provide their support and help.

Another challenge was experienced when being alone with an ill person either at home or on a walk, and being afraid that an accident would happen or that the ill person would get worse. Examples of this challenge include when a person with a balance problem went near the edge of a lake, or when a person in a wheelchair got cramps and almost fell out. However, after these experiences, the volunteers were very careful to ensure that they had the phone numbers of home nurses and relatives available, and they learned to discuss such scenarios with the relatives.

Being responsible for group meetings could be an emotional challenge, particularly since the program should be adapted to both younger and older participants at different stages of their cancer progression. The number of participants could vary from meeting to meeting; therefore, the volunteers did not know exactly who was coming. The volunteers had to be well prepared and well aware that unexpected events could happen. This was described as a balancing act, as the volunteers had to be cautious that their words did not hurt anyone who was vulnerable; at the same time, they had to be open and talk about life as it is. Likewise, they had to guide the discussion to ensure that no one in the group became too dominant at the expense of others in the group.

Their confidentiality as volunteers was unquestionable, but the volunteers had to be especially aware that that the ill individuals lived in a small place and that many individuals knew each other. Another challenge was when they were closely connected to the ill person, it could be difficult to distance themselves from that person at home. Moreover, one volunteer found it very painful to experience a conflict in a family that was not settled before the ill person passed away.

### Knowledge and life experience

To have knowledge and life experience as a health care worker was regarded as an advantage when volunteering, but it was not seen as necessary. Those who did not have such experiences were more likely to indicate that being trained was crucial, as they could obtain basic knowledge and confidence before meeting the ill persons. To be trained in communication was highlighted as necessary, as the volunteers could become aware of the importance of active listening. It was emphasized that it was important to have relational and communication skills as a volunteer, as well as to be able to speak about disease and death. To have one’s own experience with cancer, or to have family members or other relatives who passed away with incurable cancer, was stated as significant. These experiences were seen as valuable for understanding the ill persons and their relatives, and they also served to help when listening to and conversing with them, and when following the individuals in their physical and psychical states. Some volunteers stated that to have experienced illness and death in the family was a prerequisite for wanting to become a volunteer in palliative care.

### A clarified role

All of the volunteers stressed the importance of having a clearly defined role as a volunteer. This could mean that the volunteers themselves set their own boundaries and established clear agreements regarding their voluntary work, particularly when they had the time and opportunity to do so, i.e., when they wanted a sense of stability and predictability in their lives. As a part of their commitment to being a volunteer, they stated how it was important to keep scheduled appointments.

However, some volunteers who volunteered in the ill person’s home or in a nursing home, also gave more of their time than scheduled if the ill person and his/her family were in need of more help and support. Despite the fact that they wanted to be humble and let the ill persons and their families decide what type of help they wanted, the volunteers also had to clarify what they were able to do and how they wanted to be involved. For example, one of the volunteers indicated how it was important to see the ill person and her/his family, as well as the suffering they had experienced, and to be empathetic; however, a volunteer is not a part of the family, and it is the family that should be there to share the grief with the ill individuals.

When volunteering in a nursing home, it was of importance that the health care professionals were aware of the volunteer’s role. One volunteer had experienced stress among the personnel when she/he arrived to the nursing home due to the vagueness regarding the volunteer’s role. This resulted in the volunteer feeling as though she/he was perceived as controlling. Moreover, it was stated that role clarity was especially important regarding tasks that should be performed by those volunteers who had previous health care experience. There must not be any confusion between volunteering and acting as a health care professional. For example, when a nurse volunteered, she/he could not use her/his knowledge about treatments and medications.

### To be followed-up

To be followed-up continuously by a mentor with expertise in palliative care was expressed as crucial for the volunteers’ experiences. The volunteers acknowledged that they had a highly skilled mentor who knew all of them, as well as the ill persons and their families; in this way, the mentor could assign the right volunteer to the ill persons and the right volunteer to the group meetings. The volunteers acknowledged how it was highly valuable to be able to speak with the mentor after every volunteer session, especially when the volunteers were new to their role. However, after being a volunteer for a while, they no longer had the need to talk after each volunteer assignment. It was highly appreciated to know that they could contact the mentor at any time and that they could ask questions when needed, or to discuss challenging experiences, for example, on how to act when becoming deeply involved in an ill individual’s personal situation. Likewise, the mentor was able to clarify the volunteer’s role when that role was not clear to health care professionals; this was regarded as helpful.

## Discussion

The aim of this study was to describe a group of trained and supported volunteers’ lived experiences as volunteers in palliative care within the community health care services. The findings showed that the informants regarded their volunteer experiences as positive and meaningful. They prioritized being present for the ill persons throughout the course of their volunteering. However, they experienced challenges, but no one characterized these challenges as negative. They highly appreciated being trained and supported by a skilled mentor.

The findings revealed that the volunteers were very dedicated. They wanted to help and make a difference for the ill persons, and the volunteers experienced their volunteering in palliative care as positive and meaningful. One explanation might be that these volunteers were suitable for the assignment, particularly since all of them were interested in volunteering in palliative care in the first place; however, a palliative care coordinator also trained, supported, and followed-up with them very closely, which may have contributed to this experience. Claxton-Oldfield and Claxton-Oldfield [10] have stated that a volunteer coordinator’s contributions to volunteers’ experiences are positive and satisfying. Another explanation for these positive findings may be the fact that several of the volunteers had prior experience as health care personnel; thus, they had previously encountered patients with severe illness. According to Morris et al. [3], retired health care personnel often want to become volunteers. In the present study, most of the volunteers were retired health care personnel. Moreover, some of the volunteers had experience with cancer, either on their own or through a relative, which served as a valuable experience during their volunteering. To have experienced death in the family can lead to the desire to become a volunteer as well [3] which, in the present study, was also expressed as a motive by some when they decided to become a volunteer. Jack et al. [7] found similar results among volunteers in palliative care in Uganda, i.e., that they wanted to help others due to their own losses of close loved ones with AIDS.

The volunteers saw it as a privilege to be a volunteer. To mean something to someone and to make a difference for a person who was struggling was satisfying; thus, the volunteers received many benefits. They felt that they received more than they gave. Claxton-Oldfield [6] has described the benefits experienced by volunteers in palliative care, and these are concurrent with the findings in the present study, i.e., that it is a privilege to make a difference for the patient, it helps the volunteers appreciate their own lives more, and the volunteers get more from their volunteering than they give [6]. To experience satisfaction and derive benefit from their role motivates individuals to continue their volunteer activities [10]; this finding can also be applied to the volunteers in our study. The informants said that they even felt like egoists due to the fact that they wanted to continue their volunteering since they received positive feedback. One motivational factor for being a volunteer in palliative care is altruism [3, 13, 14]; however, altruism is the opposite of egoism. The volunteers did not use the word altruism, but they expressed that it was a privilege being able to help the ill persons. Since some volunteers felt like egoists, it could be interpreted that it was difficult for them to be receptive to positive feedback. To receive positive feedback can contribute to feelings of being valued [3] and to a sense of pride [7].

In the present study, the volunteers prioritized being present for each ill person throughout their volunteer experience. The volunteers stated how it was important to direct their full attention towards the ill persons in their presence, as the volunteers needed to listen and follow the ill individual in his/her physical and psychical states in order to help. This description of being present for the ill persons is similar to the concept of “true presence”, which is addressed in the nursing theory “Human Becoming” developed by Parse [15]. This theory is congruent with existential–phenomenological thoughts, such as the idea that humans engage in a mutual process with others. “True presence” is described as an attentive presence of being with another individual, and it includes being in tune with the rhythms of the human–universe process by sharing what the other wish to disclose [15]. Since the volunteers wanted to be receptive to what the ill persons wished to speak about, and given that the volunteers provided the ill persons with the space to talk about their own frustrations, can serve as an example of the volunteers’ true presence for the ill person and his/her needs. According to Parse [16], showing respect, listening intently, being with another, and sharing what this person wants to talk about are the moments where true presence unfolds. True presence with others can include face-to-face discussions with both individuals and groups [17]; this can explain why the volunteers in the present study experienced being present with the ill persons, irrespective of if they were face-to face with one individual, or if they were directly engaged with ill persons at a group level. This also confirms the idea that volunteering is relational, and how the value of volunteering rests on simply being there for the other while listening and providing support [18]. The ability to provide social support is an important task for volunteers [1, 19], and this is frequently requested by palliative patients [20]. According to Morris et al. [3] volunteers in palliative care are more empathic than other health care volunteers, which is important when providing emotional care.

All of the volunteers experienced challenges in their volunteering, but they did not experience these challenges as negative. This may indicate that the volunteers were confident in their volunteering experiences, and that they expected it might be hard to deal with disease and death. Morris et al. [3] reported that volunteers in palliative care are often emotionally robust. Some of the volunteers in the present study expressed that a volunteer cannot have mental problems, which may explain why these volunteers deemed it necessary to be emotionally robust in this role.

To have knowledge, experience, a clarified role, and to be followed-up by a mentor were continuously mentioned as crucial to the volunteers’ experiences. Clarifying the volunteer’s role is of great importance for remaining a volunteer [10] and for eliminating stressors [21]. One volunteer in the present study experienced being regarded as controlling by health care personnel. According to the findings, the mentor was able to assist the volunteers when there were misunderstandings regarding the volunteers’ roles. These findings indicate that health care personnel must be informed about the volunteers’ roles, and that a volunteer coordinator is essential for providing such education [10].

Volunteers have to be supported [22]; however, according to Horey et al. [23], no studies have provided good evidence on how to train and support volunteers in palliative care within community settings. In the present study, a palliative care coordinator had trained, supported, and followed-up with the volunteers. This coordinator possesses a unique overview and knowledge of all palliative patients and their families, which is valuable for choosing the right volunteer to work with each ill person. A skilled and charitable palliative care coordinator is thus a highly suitable individual who can be responsible for palliative care volunteers within a community setting. In the present study, the presence of a palliative care coordinator may have contributed to the successful findings. According to Morris et al. [3], volunteers’ future within palliative care is highly dependent on the relationship between the coordinator and the volunteers, as well as on how well the volunteers are supported. Claxton-Oldfield and Jones [24] also stated that the coordinator is an important person who is able to establish a good match between each volunteer and patient.

### Strengths and weaknesses

The strength of our study is that it presents trained and supported volunteers’ experiences of volunteering in palliative care settings. Such knowledge is important, since trained volunteers are desired by the community to serve as team members around palliative patients [5].

In the present study, a phenomenological research method [12] was used. In this way, the findings present the volunteers’ own experiences without interpretation. However, when analysing the interview texts, it is impossible to bracket all of one’s own knowledge, i.e., to apply phenomenological reduction, thus guaranteeing no interpretation of the findings. Nevertheless, the authors were intentionally aware that they could not use their own knowledge about volunteering in palliative care when analysing the data. Furthermore, the analysis remained true to the analysis steps, which ensures that the findings are trustworthy [25]. It should be noted that the majority of the background research performed on the topic addressed in this study was reviewed after the analysis was complete, thus preventing other studies from influencing the analysis. We can therefore argue that the findings are true to the interview texts. That other research studies also support the findings may indicate how the present findings are valid. Likewise, we can state that the general description of the phenomenon is reliable, since the same meanings were present in all interviews [26]. Thus, the general structure presents the phenomenon of “being a trained and supported volunteer in palliative care” among seriously ill or dying people. However, one can argue that the two groups of volunteers that participated in the present study may have different experiences as a volunteer, which may lead to an unclear phenomenon and a different general structure. Since the same meaning occurred in all interviews despite the fact that the volunteers performed different tasks, we argue that the essence of the studied phenomenon is clear and mirrored in an invariant general structure. According to Giorgi [12], the general structure consists of the most invariant constituents of the experience that is derived from the informants’ varied descriptions. In other words, all of the volunteers spoke about the themes included in the general structure.

Nine volunteers were interviewed in this study. It would be desirable to have more informants included in the study; however, the nine interviews provided rich data which, according to Giorgi [12], may be sufficient when there are fewer interviews. That the study group consisted of both sexes, as well as younger and older people, can be regarded as a strength, since men and younger people do not commonly volunteer [3].

## Conclusion

The findings of the present study highlight that volunteering is experienced as meaningful and satisfying, and that the volunteers have an independent and important role to play among seriously ill or dying people in the palliative care team by providing practical help and emotional support. This positive finding may be explained by the fact that the volunteers were trained and supported by a palliative care coordinator.

When volunteers experience their volunteering as meaningful, and when they receive recognition for performing voluntary tasks, they frequently wish to continue as volunteers. To ensure that palliative care volunteers are available in the future, it is important to inform individuals about the benefits of volunteering in palliative care within public forums. Likewise, health care personnel have to be aware of the volunteers’ roles in palliative care. Since palliative care coordinators are suitable for training and supporting volunteers, it is important that such a position is made available, and is filled, within the community health care services.

In future research, it will be important to focus on the experiences of patients, their next of kin, and health care personnel, particularly as they relate to the trained and supported individuals who volunteer in palliative care settings within the community health care services.
